# Effective coverage of maternal, neonatal and child health services based on District Health Information System 2 (DHIS2) data in Ethiopia: a mixed-methods study

**DOI:** 10.1136/bmjopen-2025-098795

**Published:** 2026-01-23

**Authors:** Seblewengel Lemma, Fikreselassie Getachew, Hiwot Achamyeleh, Anene Tesfa, Bantalem Yihun, Solomon Kassahun Gelaw, Theodros Getachew, Lars Ake Persson, Joanna Schellenberg, Tanya Marchant

**Affiliations:** 1 Department of Population, Policy and Practice, University College London Institute of Child Health, London, UK; 2 Health System and Reproductive Health Research Directorate, Ethiopian Public Health Institute, Addis Ababa, Ethiopia; 3 Ethiopia Ministry of Health, Addis Ababa, Ethiopia; 4 Global Health and Population, Harvard T H Chan School of Public Health, Boston, Massachusetts, USA; 5 Disease Control, London School of Hygiene and Tropical Medicine, London, UK

**Keywords:** Ethiopia, Feasibility Studies, Health Services Accessibility, Quality in health care, Quality Improvement

## Abstract

**Abstract:**

**Objective:**

Our objective was to assess the feasibility of using the routine health information system data source, District Health Information System (DHIS2) to measure the effective coverage of selected health service indicators in Ethiopia and to explore stakeholder perceptions of those measures.

**Design:**

We conducted a mixed-methods study. We mapped the availability of data elements in DHIS2 between July 2022 and June 2023 for five indicators (four or more antenatal care visits (ANC4+), skilled birth attendance, postnatal care, sick child care and child nutrition care). We defined effective coverage cascade steps for each indicator, assessed data quality and analysed data using STATA V.17. Finally, qualitative interviews were conducted with 15 key stakeholders, and the data were analysed thematically for reflections on the DHIS2 output.

**Setting:**

The data were captured from all public health facilities of 11 regions and 2 administrative cities in Ethiopia.

**Results:**

There was better availability of data elements for maternal healthcare than for child healthcare. It was possible to estimate the intervention-adjusted coverage of ANC4+ (16% nationally) and the process-quality-adjusted coverage of skilled birth attendance (19% nationally). Postnatal care, sick child care and child nutrition indicators lacked data across multiple cascade steps. The quality of data for effective coverage measurement differed by region. The key informants expressed concerns about the adequacy and appropriateness of DHIS2 data for this analysis. While all acknowledged its potential for decision-making, respondents emphasised the need for standardised methods and data sources to enhance comparability and acceptability of the findings.

**Conclusions:**

The findings underscore the need for system-level improvement of data availability and quality, and adoption of a standardised approach to calculating effective coverage using DHIS2. There was a concern that the findings may not be accepted by policymakers; however, the local level granularity made possible through DHIS2 was appreciated.

**STRENGTHS AND LIMITATIONS OF THIS STUDY**

- The study provides a rigorous assessment of District Health Information System-2 (DHIS2) for the purpose of effective coverage measurement in Ethiopia, highlighting strengths and weaknesses of the data source.
- Using a cascade approach, DHIS2 data elements were mapped, and indicators analysed across the maternal, newborn and child health continuum.
- Results also include the qualitative responses of key stakeholders to the quantitative analysis, highlighting perceived areas of strength and weakness.
- Private health facilities were not included in the analysis because of their lack of data in essential data elements and indicators.
- The definition of effective coverage cascade steps was largely influenced by the availability of data elements to illustrate what was possible in the existing system.

## Introduction

 A well-functioning health system strives to provide quality health services accessible to all population segments according to their needs. As such, appropriate measures of quality health services provide useful information for decision-making that supports improvement.^1 2^ Without measuring quality, the effect of a health service on outcomes is not predictable.^3^,^5^

A growing number of studies and initiatives attempt to combine metrics on health service access with service quality dimensions, showing large gaps between crude and adjusted measures,^6^,^11^ implying that many people access health services of suboptimal quality.^12^ But there is a lack of consistency between approaches and the need to make effective coverage measurement more routine has been identified.^13^ This need has been expressed in at least two ways. First, there is a need to track the overall health system performance toward achieving universal health coverage and guiding further policy formulation. Second, there is a need to guide individual patient care and monitor health facility-level quality improvement initiatives.^14^

Barriers to meeting these needs include challenges in choosing suitable data sources. Effective coverage measures are data-intensive since they include multiple dimensions of health service provision.^12 15^ Many studies have linked retrospective population-based sample surveys with health facility sample surveys. Such studies are frequently relied on for benchmarking coverage and tracking progress. However, their periodic design is not ideal to inform the everyday efforts of health facilities and local administrators.^13 16^

Conversely, routine health information system (RHIS) data, including the District Health Information System-2 (DHIS2), have the potential to summarise efforts at very granular levels and in real time.^2 17^ To date, their use in measuring effective coverage has been limited, mainly due to concerns about suboptimal data quality. However, there are increasing calls to strengthen RHIS data quality and address this underutilisation problem.^18^,^20^

Ethiopia is a country that prioritises both effective coverage measurement and the usability of routine health data. Recognising the potential utility of effective coverage measures to support improvements in health system quality, the Ethiopian Ministry of Health (MoH) drafted a guideline in 2024 to use effective coverage measurements to monitor the performance of the health system for a range of health services, including maternal, neonatal and child health (MNCH) services. The country has also invested in efforts to strengthen the quality and use of the RHIS. In this study, we evaluated the potential to bring these two priorities together. We specifically assessed the feasibility of using DHIS2 data to estimate effective coverage for selected MNCH indicators and explored key stakeholders’ perceptions of effective coverage measures derived from Ethiopian DHIS2 data.

## Methods

### Study setting

Ethiopia is divided into 12 regional states and 2 administrative cities (Addis Ababa and Dire Dawa). With a current population of more than 100 million people, Ethiopia has substantially improved access to essential health services for mothers, newborns and children.^21^ The MoH has developed strategies and policies to improve RHIS data availability and use, using DHIS2 as the main platform.^22^ Managed through an executive office within MoH, data from all health facilities in the country are collected and reported with an inbuilt dashboard that allows analysis and data visualisation at the health facility, district, regional and national levels.

### Use of a standard effective coverage cascade

Effective coverage measures for MNCH are formulated using a health service coverage cascade of distinct steps (online supplemental figure 1). For MNCH care, which involves complex interventions with multiple components at each step, the recommendation is to proceed to the process quality-adjusted step.^12^ The target population is defined as a population with specific health needs. Service contact coverage is the proportion of the target population that accesses the relevant health service. Input-adjusted coverage is the proportion of the target population accessing health services that are ready to provide this care. Intervention-adjusted coverage is the proportion of the target population who access services prepared to provide care and receive the appropriate interventions. Process-quality-adjusted coverage is the proportion of the target population who access health services ready to provide care, where appropriate interventions are delivered, and services are provided according to quality standards.^12^

### Study design

Our study was implemented in three stages: (1) mapping the availability of relevant data elements in DHIS2 for estimating effective coverage, (2) analysing those DHIS2 data elements as effective coverage indicators, and finally, (3) conducting and analysing qualitative interviews with key stakeholders in Ethiopia about the utility of effective coverage measures generated using DHIS2.

#### Mapping of data elements in DHIS2

Five MNCH services were identified for effective coverage measurement^14 23^: at least four antenatal care contacts (ANC4+), skilled birth attendance, postnatal care (PNC), sick child care (care for fever, pneumonia and diarrhoea) and child nutrition service (nutritional screening, diagnosis and treatment of severe acute malnutrition). We reviewed the national guidelines for these services,^24 25^ identified their individual service components and the inputs needed to provide them and created a data abstraction form to extract the identified data elements from the DHIS2 platform. Details of the data elements and definitions of the five core interventions are presented in table 1.

**Table 1:** Measurement, definition and available data elements in DHIS2 to calculate effective coverage of ANC4+, skilled birth attendance, PNC, sick child care and child nutrition service

Steps	ANC4+	Skilled birth attendance	PNC	Sick child care	Child nutrition service
Target population	All pregnant women in a catchment =number of pregnant women recorded in DHIS2 to have come to a facility for ANC1 divided by the population-level proportion of pregnant women who reported to have at least one ANC*	All deliveries in a catchment =estimated number of pregnant women (as for ANC) multiplied by (1–0.03) to account for pregnancy loss* For newborn interventions: total live birth reported in the DHIS2 in the same period	As for skilled birth attendance	No data elements were available in DHIS2 to estimate the target population ‘All under-5 children in need of care for illness’	No data elements were available in DHIS2 to estimate the target population ‘All under 5 children who seek care in a health facility’
Contact coverage	% pregnant women with ANC4+ visit during 2022/2023 Numerator=number of pregnant women with ANC4+ Denominator=target population	% of pregnant women who delivered at health facilities Numerator=number of pregnant women who were assisted by skilled attendant during childbirth Denominator=target population	% of PNC in the first 7 days postpartum Numerator=number of postnatal mothers and their newborns who received PNC within 7 days Denominator=total live birth	% of target population who visit health facility for symptoms of pneumonia, diarrhoea or fever Numerator=DHIS2 only provides the number of episodes of the illnesses Denominator=not available	% of target population who visited health facility for nutrition service Numerator=number of children screened for malnutrition (proxy for contact) Denominator=not available
Input coverage	% of health facilities that meet the conditions for infrastructure and staff and drugs	As for ANC	As for ANC	As for ANC Note: with a different list of tracer drugs specific to sick child care (11 drugs for health centres and hospitals and 6 drugs for health posts)	As for ANC Note: list of drugs didn’t include items needed for nutrition service
	Infrastructure % of health facilities that have at least one of the infrastructure items **:** sanitation facility, electricity, water				
	Staff % of health facilities that fulfilled 50% or more of the recommended staffing standard				
	Drugs % of health facilities with 50% or more of 22 listed essential drugs (online supplemental table 4)				
Intervention coverage	Average coverage among ANC attendees of the four interventions**:** tetanus-diphtheria 1 vaccination, iron folate distribution, being tested for hepatitis B and syphilis	% of mothers who received uterotonics right after birth Numerator: number of mothers who received uterotonics Denominator: total birth % of newborns who had chlorhexidine for cord care Numerator: number of newborns who had chlorhexidine Denominator: total live birth	No intervention data element available in DHIS2	No intervention data element was available	Possible data elements in DHIS2 were screened for malnutrition, diagnosed and received treatment based on malnutrition status.
Process quality coverage	No process quality data elements available in DHIS2	% of newborns for whom their weight was measured Numerator: number of newborns weighed Denominator: total live birth	No process quality data elements available in DHIS2	Possible data elements in DHIS2 were assessed for developmental milestones, growth monitoring and screening for acute malnutrition	No process quality data available in DHIS2

* See reference 27.

ANC4+, four or more antenatal care visits; DHIS2, District Health Information System; PNC, postnatal care.

#### Effective coverage analysis using DHIS2 data elements

We accessed 1 year of data from DHIS2 from July 2022 to June 2023 in the Gregorian calendar (corresponding to the Coptic calendar 2015 fiscal year in Ethiopia). All regions of the country were included except Tigray, which lacked data due to postinternal conflict. All public health facilities were included, representing the major healthcare provider in Ethiopia. Private health facilities were excluded due to their limited contribution of data to DHIS2 in Ethiopia. Data were imported to Stata V.17 for cleaning and analysis. In total, we analysed data from 4015 health facilities: 3638 health centres, 252 primary hospitals, 94 general hospitals and 31 referral hospitals.

A simple descriptive analysis was conducted for each data element to explore the data and check data quality. Reporting completeness was calculated as the percent of reports available during the 1-year reporting period for each data element included in the analysis,^26^ and results were compared across regions (see online supplemental tables S1–S3). In our analysis, all missing data were treated as none and were replaced with zero.

Based on the data elements that were available in DHIS2, we developed definitions of effective coverage and cascade steps, presented below as results. Available data elements meant that it was possible to construct effective coverage for ANC4+and skilled birth attendance, and a detailed checklist for these indicators is presented in online supplemental tables 4 and 5.

#### Qualitative interviews with key stakeholders

We invited 15 key informants to comment on the results, eight from MoH and seven from partner organisations. These informants were purposefully selected using a snowball sampling technique, ensuring they had significant expertise or familiarity with tracking the quality of health services, particularly in MNCH.^14^

The research team (SL, HA, AT and FG) conducted the interviews using a guide prepared in English and translated into the local language, Amharic (online supplemental box 1). To maintain consistency, the guide was back-translated into English. The guide was pretested with a small sample to assess the clarity of the questions and the overall interview process.

Due to logistical constraints, two interviews were conducted via online conferencing software, while the remaining interviews were carried out in person. On average, each interview lasted 60 min. All interviews were tape-recorded, and detailed notes were taken during each interview. The transcription process was conducted in English simultaneously with the interviews to ensure the accuracy and immediacy of data capture. The final sample size was determined based on saturation when no new information was generated from the interviews.

For the qualitative analysis, we imported all transcripts into NVivo V.12. The research team used an inductive method to develop the code book, collectively coding one transcript, followed by a discussion to agree on the codes used across all interviews. The four interviewers, splitting the transcripts among themselves, did the coding for all interviews (SL, AT, HA and FG). The coded transcripts were read and re-read to identify patterns and themes. These patterns and themes were subsequently shared with the key informants in a consultative workshop designed to test our understanding of participant views and to validate the findings, ensuring that the interpretations were aligned with the informants’ perspectives.

### Patient and public involvement

None.

## Results

**Figure 1:**
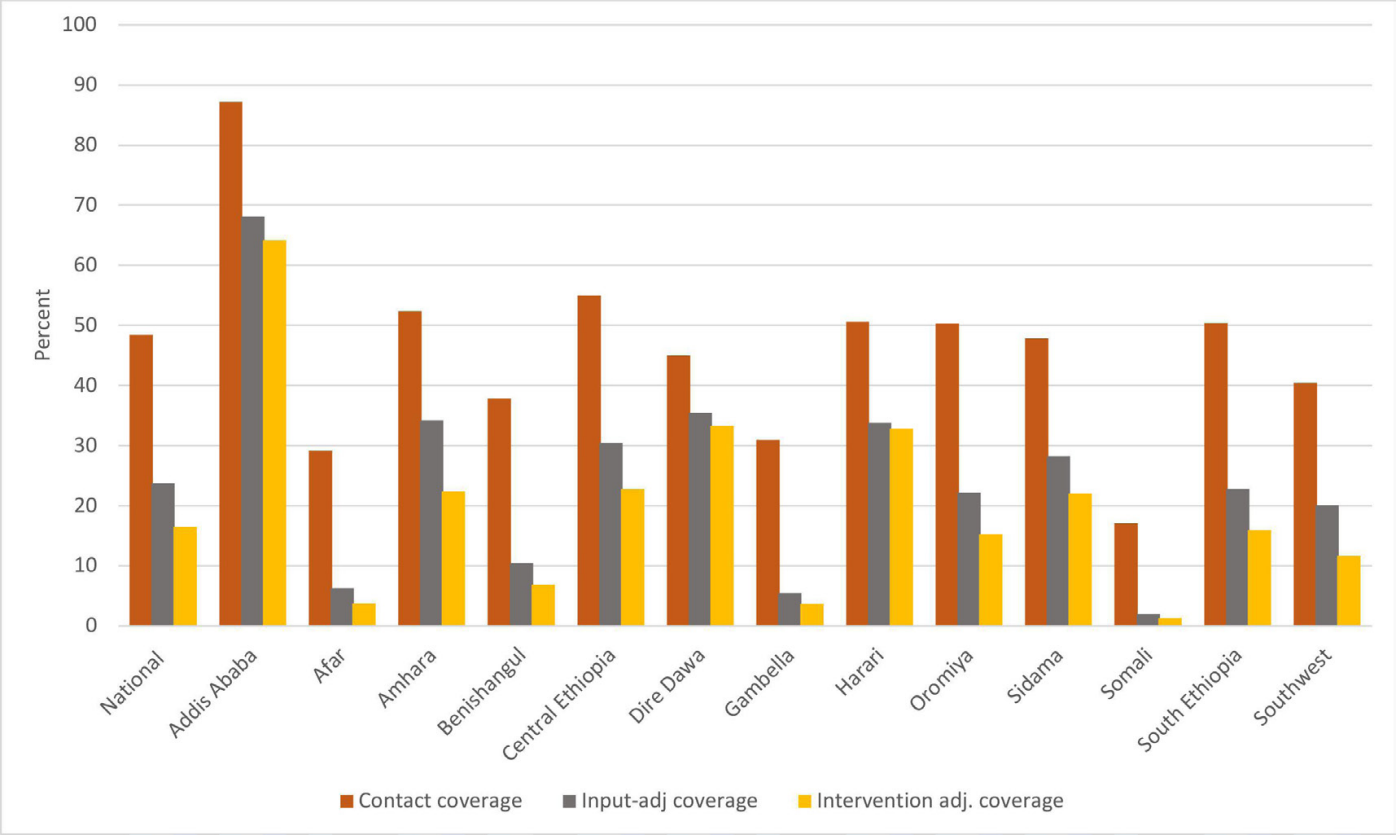
National effective coverage cascade for four or more antenatal care visits, disaggregated by region. Data from Ethiopian District Health Information System 2 July 2022–June 2023.

Below, we summarise findings on (1) DHIS2 mapping for each indicator in turn (table 1) and, where relevant data elements are available for an indicator, (2) present effective coverage analysis (figures1  2). Finally, (3) we present the perceptions of key stakeholders about these findings.

**Figure 2:**
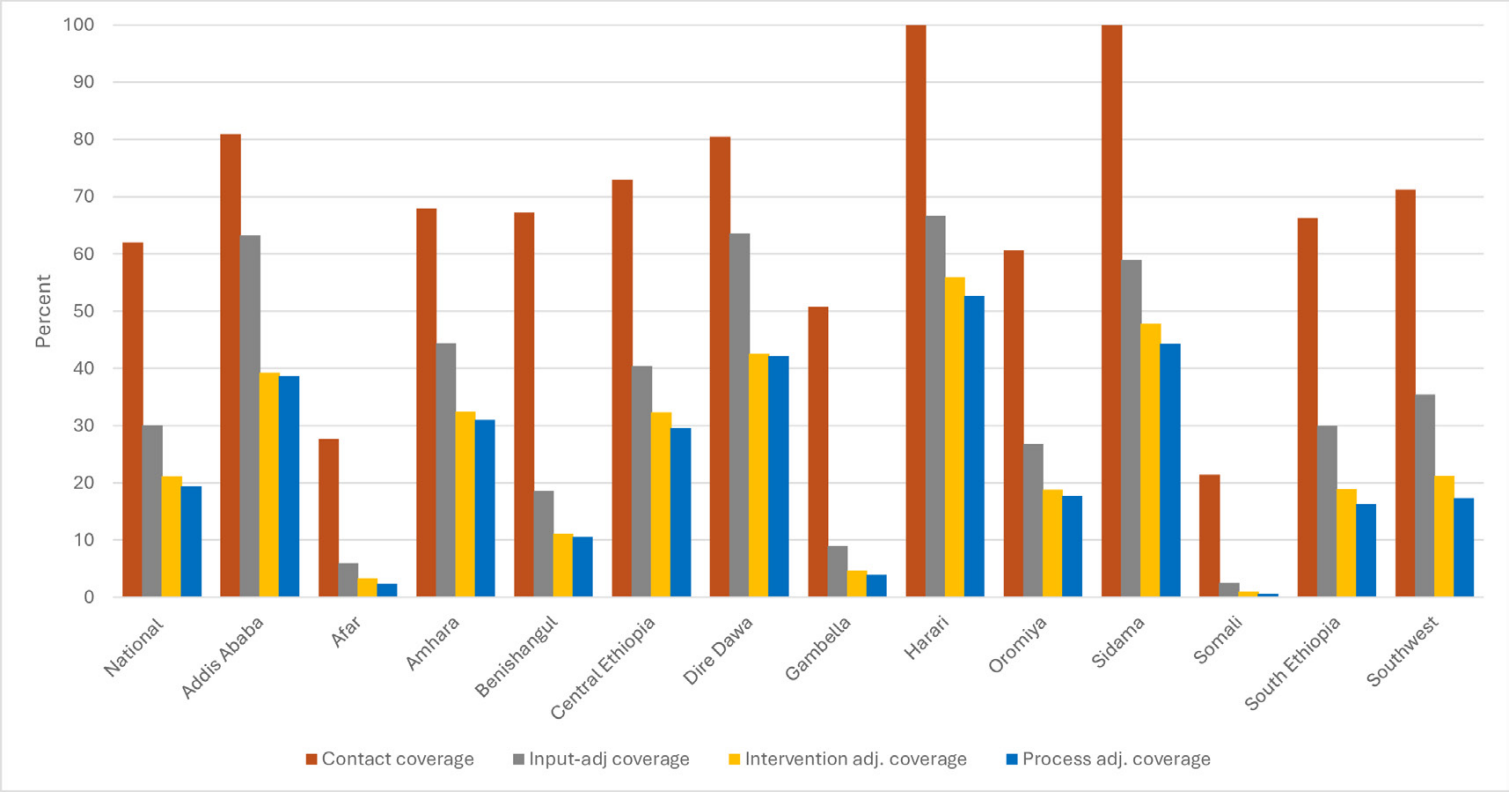
Effective coverage cascade for skilled birth attendance by region. Data from Ethiopian District Health Information System 2 July 2022–June 2023.

### ANC coverage

#### Mapping available data elements

The data elements available in DHIS2 to construct an effective coverage measure for at least four ANC visits in Ethiopia resulted in an intervention-adjusted indicator defined as *“the proportion of pregnant women who, at least four times during pregnancy, accessed a health facility with adequate inputs (electricity, sanitation, water supply, and both staff and essential drugs), who received appropriate interventions (iron-folate, Tetanus-Diphtheria-1 vaccination, tests for syphilis, and hepatitis B)”*. No data elements were available to extend the effective coverage measure to include process-quality-adjusted coverage (online supplemental table 4).

Target population: The target population represents the denominator for the effective coverage cascade. In DHIS2, a count of the number of pregnant women making at least one ANC visit is reported monthly, but no population-level denominator is available to represent the target population. As a proxy, we implemented the method recommended by Countdown2030^27^ of dividing the DHIS2 count of pregnant women who accessed at least one ANC visit by the coverage of at least one ANC as reported from the most recent Ethiopian Demographic and Health Survey.^27^

Service-contact coverage: The number of pregnant women who received at least four ANC visits is reported monthly and was used as the numerator to estimate contact coverage.

Input-adjusted coverage: Following our review of Ethiopian guidelines (national indicator reference, ANC, skilled birth attendance and PNC guideline),^23^,^2528^ three input categories were defined.

For health infrastructure*,* three data elements were reported annually by each health facility as ‘yes’ or ‘no’: the availability of electricity, sanitation facilities and water supply. We defined health infrastructure to have at least one of the three infrastructure elements.

For human resources, DHIS2 provided data elements at the level of the health facility on the number of health workers who were (1) employed at the beginning of the year, (2) recruited during the year and (3) left the facility at the end of the year, disaggregated by cadre. To construct an estimate of human resources, we took the number of nurses, midwives and health officers available in each facility at the beginning of the year and related this to the MoH staff number requirement for each health facility level. Health facilities that fulfilled at least 50% of the requirements for the selected health worker categories were regarded as having adequate staff to provide the services.

For commodities*,* data elements were available for essential drugs. In DHIS2, the availability of each essential drug was reported monthly as ‘yes=available’ or ‘no=not available’. For each health facility, we counted the number of tracer drugs available over the 12-month period and divided it by the total number of tracer drugs that were expected to be available in 12 months. We categorised the health facilities as having at least 50% of the tracer drugs during the reporting period or fewer than 50% of the tracer drugs.

Finally, each health facility was categorised as a ready facility if it had the infrastructure, health staff and commodities as defined above and percent availability was presented by region (table 2).

**Table 2:** Availability of input data elements (infrastructure, human resources and essential drugs) for hospitals and health centres, for antenatal care and skilled birth attendance, Ethiopia July 2022–June 2023

Region	Health infrastructure %	Human resource %	Tracer drugs %	Input coverage %	Number of health facilities
Addis Ababa	91	81	98	78	105
Afar	46	28	74	22	111
Amhara	94	67	98	65	972
Benishangul	50	30	55	28	76
Central Ethiopia	86	56	95	55	255
Dire Dawa	89	79	95	79	19
Gambella	26	21	71	18	34
Harari	92	67	92	67	12
Oromia	82	48	87	44	1565
Sidama	88	61	94	59	161
Somali	39	14	63	12	246
South Ethiopia	75	50	85	45	314
Southwest	80	57	87	50	145
National	80	52	88	49	4015

Intervention coverage: To estimate intervention coverage, four interventions in Ethiopia’s ANC guidelines were present in DHIS2 and were selected as tracer interventions: provision of iron and folate supplements, tetanus-diphtheria 1 vaccine, tests for syphilis and tests for hepatitis B. These were presented as counts of women who received each intervention. To construct an indicator of intervention coverage, we took the average coverage of the four tracer interventions (table 3).

**Table 3:** Maternal and newborn health access and intervention coverage in Ethiopia, July 2022–June 2023*

Regions	ANC4+ and related services						Skilled delivery and related services					PNC
	ANC 4+ contact coverage %	Tested for Hepatitis B %	Tested for Syphilis %	TD1 %	Iron folate %	Average intervention coverage %	Skilled delivery contact coverage %	Uterotonics %	Chlorohexidine %	Average intervention coverage %	Process: Newborn weighed %	Early PNC contact coverage %
Addis Ababa	87	98	99	89	92	94	79	97	27	62	99	85
Afar	29	50	57	63	68	60	27	87	25	56	69	30
Amhara	52	82	91	4	85	65	66	99	48	73	95	64
Benishangul	38	61	68	54	80	66	65	92	29	60	94	58
Central Ethiopia	55	81	98	39	82	75	71	95	66	80	92	57
Dire Dawa	45	100	100	48	97	94	78	100	32	67	99	72
Gambella	31	54	61	80	77	68	49	100	5	52	84	49
Harari	51	87	93	69	100	97	98	100	64	84	94	87
Oromia	50	70	87	40	77	69	59	98	43	70	94	60
Sidama	48	59	100	57	85	78	100	96	65	81	93	86
Somali	17	71	77	50	60	64	21	67	11	38	59	44
South Ethiopia	50	75	88	37	79	70	64	91	35	63	86	27
Southwest	40	44	60	56	72	58	69	91	29	60	81	67
National	48	74	88	36	80	70	60	96	44	70	92	62

* Percentages derived from numerators based on DHIS2 counts and denominators based on DHS 2019.

ANC, antenatal care; DHIS2, District Health Information System; PNC, postnatal care.

Process quality coverage: No data elements for ANC4+process indicators were available in DHIS2.

#### Data quality

We checked the reporting completeness of the data elements and recognised variation in data quality by indicator as well as by region (online supplemental tables S1–S3). At the national level, health facilities had over 90% completeness of ANC1, ANC4+ and for tracer drugs. But data completeness was much lower for elements related to infrastructure and human resources. And large variations were observed in reporting completeness between regions (online supplemental table S1).

#### Analysis of the effective coverage of ANC4+

The target population was estimated to be 3.9 million in 2022/2023. Based on the DHIS2 data, the percent of the target population having at least four ANC visits was 48%, ranging from 17% in Somali to 87% in Addis Ababa (table 3).

The overall input coverage, that is, the proportion of health facilities reported to have all three categories of inputs, was 49%, ranging from 12% in Somali region to 79% in Dire-Dawa (table 2). The average intervention coverage of the four interventions was 70%, ranging from 58% in Southwest Ethiopia to 97% in Harari (table 3).

Overall, 16% of pregnant mothers were estimated to have received intervention-adjusted ANC4+, being less than 5% for Afar, Gambella and Somalia, while Addis Ababa had the highest performance (64%).

### Skilled birth attendance

#### Mapping available data elements

The data elements available in DHIS2 to construct an effective coverage measure for skilled birth attendance in Ethiopia resulted in a process-adjusted indicator defined as *“the proportion of the pregnant population who accessed a health facility for childbirth care where there were adequate inputs (electricity, sanitation, or water supply, where staff and essential drugs were present), who received appropriate interventions (uterotonics immediately after birth and chlorhexidine for the cord care) and according to the quality standard (newborn weighed)”*.

Target population: a denominator was needed for adjusted measures of quality care for mothers plus—for example to estimate the access and subsequent quality steps, and a denominator was needed for adjusted measures of quality care for newborns—for example, to estimate coverage of chlorhexidine application and baby weighed. We estimated the expected number of mothers by multiplying the total number of pregnancies by 97%, accounting for 3% pregnancy loss.^27^ We estimated the number of newborns from the total number of live births during the same period reported in DHIS2 (online supplemental table 5).

Contact coverage: The total number of births attended by skilled personnel was reported monthly in DHIS2 and was used to calculate contact coverage.

Input coverage: Same as for ANC4+.

Intervention coverage: Two interventions that are required for all women and the newborn were available in DHIS2: administration of uterotonics to the mother immediately after birth and the application of chlorhexidine on the newborn baby’s cord.

Process quality coverage: Weighing of the newborn was included as a process quality data element. No other data elements were available.

#### Data quality

The reporting completeness for data elements specific to skilled birth attendance was above 82% except for chlorhexidine (44%). Large regional variations were also noted (online supplemental table S2).

#### Analysis of effective coverage of skilled birth attendance

The estimated number of deliveries for this period, the target population, was calculated to be 3 783 000. Based on the DHIS2 data, the percentage of the target population to have received skilled birth attendance was 62% ranging from 21% in Somali to 100% in Sidama and Harari (table 3). Input coverage, which included all items and assumptions made for ANC4+ analysis, was 49%. Uterotonics were given to most women (96%) and 44% of the newborns had chlorhexidine applied to the cord. Thus, the average intervention coverage was 70%, ranging from 38% in Somali to 84% in Harari. On average, 92% of newborns were weighed (table 3). The overall process quality-adjusted coverage of skilled birth attendance was 19%, ranging from 1% in Somali region to 53% in Harari region (figure 2).

### Postnatal care

#### Mapping available data elements

According to the national indicator reference guide, receiving PNC is defined as getting at least one postnatal visit within 7 days after delivery. Data elements were available for the target population, contact coverage and input adjusted coverage and are described below. But no elements were available to continue further along the cascade and consequently, no effective coverage analysis was carried out.

Target population: The denominator could be calculated as for skilled birth attendance (above).

Contact coverage: This was available from the DHIS2 data element as PNC within 7 days of delivery.

Input coverage: The same data elements and assumptions could be used as for ANC4+, which included items relevant during PNC, for example, iron folate, tetracycline eye ointment, gentamicin and amoxicillin dispersible tablet.

Intervention coverage: There were no data elements for PNC interventions.

Process quality coverage: No data elements for PNC process quality.

### Sick child care

#### Mapping available data elements

We considered the care provided to sick children for three common childhood illnesses: diarrhoea, pneumonia and fever as depicted in DHIS2. However, given data limitations mentioned below, including for the target population, we were not able to calculate effective coverage for sick child care.

Target population: No data element was identified that could estimate the number of children under 5 who were in need of sick child care at the population level.

Contact coverage: DHIS2 provides data on the total number of visits to health facilities by under-5 children. As a count of the number of visits, it reflects the total number of illness episodes rather than the total number of children receiving care.

Input coverage: It is possible to calculate the percentage of health facilities that had essential inputs available for sick child care (eg, using the MoH list of essential drugs, as done for ANC4+, plus staff lists and infrastructure). However, in the absence of a target population, it was not possible to calculate input coverage within DHIS2.

Intervention coverage: No data elements were available that distinguish specific treatments to a diagnosis for childhood illness in DHIS2. Rather, DHIS2 records the aggregate number of children who were treated for an illness.

Process quality coverage: Screening for malnutrition could potentially be used as a process quality indicator in DHIS2 if appropriate denominators were available.

### Child nutrition service

#### Mapping available data elements

As for sick child care, because of limited data elements in DHIS2, it was not possible to measure the effective coverage of child nutrition services, including estimating the target population.

Target population: The target population should be children under 5 years in need of nutrition services, but no data source was identified with which to estimate this number.

Contact coverage: As in the target population, no data were available in DHIS2.

Input coverage: The input data elements used in the earlier analysis lack tracer items such as the availability of nutrition supplements and other inputs needed to provide the service, making it inadequate for estimation of facility readiness to provide nutrition services.

Intervention coverage: Some interventions were reported through DHIS2, such as screening for acute malnutrition, diagnosis and treatment that could be used to do the analysis if calculating the prior steps in the cascade was possible.

Process coverage: There were no data elements used as process quality indicators such as counselling in infant and young child feeding.

### Stakeholder feedback

The results of the mapping and analysis of DHIS2 data for effective coverage measurements were presented to stakeholders and their responses are summarised below under three themes: reflections on DHIS2 for effective coverage measures, the need for harmonisation in effective coverage indicators, and the potential utility of the measures.

#### Reflections on DHIS2 for effective coverage measures

Three main reflections emerged: (1) that the ambition to use DHIS2 to measure effective coverage was appreciated, but (2) more data elements were needed and (3) DHIS2 data quality needs continuous improvement.

Regarding ANC and skilled birth attendance, for some participants, the findings accurately depicted the on-the-ground reality.

“It’s not surprising at all because, for a long time, we have focused solely on monitoring coverage and contact. This necessitates a paradigm shift…Therefore, I can confidently say that while not unexpected, these results are indeed disappointing.” (Key informant from partner organization)

Respondents would have preferred more data elements to have been included if available in DHIS2. The need to address complications, for instance, during skilled birth attendance was emphasised.

The lack of process quality data elements was a particular concern, and participants recommended triangulating RHIS data sources to extend the cascade up to the process-adjusted coverage level.

“For instance, when we speak about ANC, you solely address the intervention and leave out the process, even though the process’s components are crucial*.”* (Key informant from partner organization)

For sick child care and child nutrition, no source of denominator data was identified.

“The question is without determining the target population can we go to the next step in the cascade…. It also questions how much DHIS2 can be trusted.” (Key informant from MoH)

Overall, the use of DHIS2 for effective coverage measures was thought to hinge on the quality of the data, with improved data quality being a prerequisite for using findings to inform health service quality improvement.

“One of the issues that affect usability is the data quality… If the data quality is ok, you can trust the data. It is crucial to work on the data quality at the health facility and the needed data should come to the central for the effective coverage analysis. I recommend the use of DHIS2 data.” (Key informant from partner organization)

#### The need for harmonisation of indicators

The participants recognised the need to compare results within a country and beyond. They noted that while the methods used had been straightforward, the findings were not comparable with other studies conducted in Ethiopia and elsewhere, primarily due to the data elements available in this data source. While it was appreciated that MoH guidelines had been used as a starting point, it was observed as a problem that the final effective coverage definitions were driven by the data elements available.

For the input data, one participant said:

“In terms of the method, DHIS2 by itself restricted your work. I observed a study on the effective coverage of sick child care in Tanzania, which included process quality measures. And I fear that it will be challenging to contrast your results with previous discoveries. Given the limitations of your results, this will be a hurdle.” (Key informant from MoH)

Respondents sought to have a clear recommendation on how to define each cascade step with available data, and suggested further discussion and standardisation of indicator definitions.

#### Utility of the results

Respondents agreed that the results were useful at different levels of the health system. For some, the usability was more prominent at the national and regional levels, as it enabled regular evaluation of health system performance and could guide improvements of health system building blocks.

“The extent may differ, but I believe this would be helpful for caregivers in the health facility up to the policymakers. The way they will be informed could be different, but it helps all*.”* (Key informant from MoH)

Others suggested the use of such results at the national level only because many of the changes needed were structural.

“The first level is at the ministry level; it reveals our gaps and areas we should prioritize and most of the result at this level pertains to inputs…” (Key informant from MoH)

For others, results were directly related to health service provision in health facilities. They strongly advocated for such analyses to occur within health facilities, with quality improvements commencing at that level.

“The health facilities benefit from this kind of result. To be honest, we have invested a lot in the health sector for the last many years, and we need to do our investment in a way that profits the community, and we have to monitor if this investment brought change*.”* (Key informant from partner organization)

Participants were asked about the frequency of conducting such analyses. Those advocating for the utilisation at the lower levels recommended quarterly to bi-annual analysis to drive improvements in health service quality at the grassroots level. For national-level utilisation, an annual analysis was suggested. Additionally, one participant proposed the use of surveys instead of DHIS2 data and recommended conducting surveys every 2 years.

Finally, some participants were concerned that effective coverage results may not be acceptable to decision makers, including policymakers. Because the adjusted coverage results were much lower than the crude coverage, which could be both unexpected and shocking. As a remedy, participants suggested providing a clear and detailed description of the method used along with the result, to improve users’ understanding.

“In my opinion, you used the proper methodology, so there is nothing controversial about it. However, when you go to policymakers, they may not accept this easily due to various reasons.” (Key informant from partner organization)

## Discussion

We assessed the feasibility of using DHIS2 data for estimating effective coverage of ANC4+, skilled birth attendance, PNC, sick child care and child nutrition in Ethiopia. Generally, there was better availability of data elements for maternal healthcare than for child healthcare. For example, we were able to estimate the intervention-adjusted coverage of ANC4+, being 16% nationally in this example. And it was also possible to estimate the process-adjusted coverage of skilled birth attendance, being 19% nationally. However, PNC lacked data elements for both intervention and process quality, and the sick child care and child nutrition indicators lacked data across multiple cascade steps, including the target population. Presented with this information, key informants expressed concern about the adequacy and appropriateness of using DHIS2 for effective coverage analysis in Ethiopia, citing issues with data quality and inadequate data elements for some of the cascade steps. While all acknowledged the potential value of DHIS2 for decision making at national, regional and facility levels, they emphasised the need for a standardised method and data source to enhance comparability and acceptability of quality-adjusted findings.

This is the first study to assess the feasibility of using DHIS2 for the purpose of generating key effective coverage indicators in Ethiopia. Two previous studies have reported use of RHIS data in combination with population surveys for effective coverage. In Nigeria, DHIS2 was used to measure effective coverage of childbirth care up to the intervention-adjusted coverage step; and in Mexico, a routinely collected performance indicator was used to report effective coverage for a number of indicators, although the standardised effective coverage cascade was not applied.^7 29^ There is a clearly expressed need for more country examples of what is possible so that harmonised recommendations can be generated to guide action on effective coverage.

We used qualitative methods to provide the perspective of experts and end users of effective coverage indicators. Four main issues emerged around the suboptimal availability of data and data quality, summarised below.

First, a major issue was the inability to estimate the denominator or target population for child health indicators. Ethiopia’s DHIS2 relies on census data to estimate the size of key target populations. The latest Ethiopian census was conducted in 2007; even with adjustment factors, such estimates may not be reliable.^20 30^ An alternative approach was to use information on access to care from a recent population survey to estimate the target population, as we did for maternal indicators,^27^ although no access indicators reached 100% of the population in need. Furthermore, for child health, DHIS2 records the number of illness episodes, or the number of visits made by under-5 children, rather than the number of children being served, making it impossible to combine with survey data to calculate the denominator.

Second, the lack of data elements across quality dimensions influenced definitions of cascade steps, sometimes making them unsatisfactory. Data elements associated with process quality (eg, around counselling or respectful care) were missing for nearly all services, even though these data points were sometimes available in facility registers. This limited availability of data elements for process quality was also observed in the Nigerian study that assessed the feasibility of using a routine system to estimate effective coverage^7^ and in a study that assessed the feasibility of using WHO quality indicators for maternal and newborn care.^31^

Third, defining cut-off values for inputs such as infrastructure was a challenge, partly because of the way that missing data is handled in DHIS2. Data elements in DHIS2 may lack a value because the item is not available in a given health facility or because the data is missing: DHIS2 records both scenarios as missing. This lack of transparency in the data slowed progress in our analysis and led to the team making the pragmatic decision to set required availability of these items at 50%. This was not intended as a definition recommendation but does highlight the need for global or country level recommendations on how to handle such limitations.

Finally, data quality was a concern, with large differences observed between regions and by indicator. This lack of consistency further limited end-users’ trust in data outputs. Similar observations were reported in previous Ethiopian studies^20 32^ and elsewhere.^33 34^ Better data quality was observed for maternal than for child health services. This difference could be a result of more targeted improvement efforts and significant focus on maternal health services by MoH. The regional differences could likely reflect the variation in the state of health system establishment across the regions, also illustrated by the regional variation in the state of health in Ethiopia.^35^ The disparities in data quality imply that data quality improvement is feasible. Furthermore, data that were collected less frequently (eg, annually for infrastructure and human resource data) had markedly lower quality due to fewer quality checks.

This study has some limitations. As for all effective coverage measurement that uses aggregated health facility data, the effective coverage estimates based on DHIS2 may not reflect an individual’s experience with the health service. The findings are most relevant to countries using the DHIS2 platform. We excluded the private health facilities because of their lack of data on essential data elements and indicators: while this limitation was acceptable for Ethiopia, where a large share of healthcare is provided by the public sector, it may have limited generalisability to countries where private health facilities play a larger role. Crucially, our definitions of effective coverage cascade steps were largely influenced by the availability of data elements and illustrate what was possible to measure using the DHIS2 data but should not be seen as recommendations.

## Conclusions

Our analysis of effective coverage using DHIS2 data highlighted both the possibilities and the challenges that may arise. The findings underscore the need for system-level improvement in data availability and quality for effective coverage, as well as the adoption of a standardised approach. The local level granularity of the data was much appreciated and could improve the quality of individual patient care at the health facilities as it allows for better insight to more specific issues in patient care, contributing to improvement in health service quality at the district level and beyond.

## Supplementary material

10.1136/bmjopen-2025-098795 online supplemental file 1

## Data Availability

Data are available on reasonable request.
