# Supplementary material for: Effective coverage of maternal, neonatal and child health services based on District Health Information System 2 (DHIS2) data in Ethiopia: a mixed-methods study
Source: BMJ Open. 2026 Jan 23;16(1):e098795. doi: 10.1136/bmjopen-2025-098795 (PMC12853494; doi:10.1136/bmjopen-2025-098795)
Supplement: online supplemental file 1 [file bmjopen-16-1-s001.docx]

**Supplementary materials**


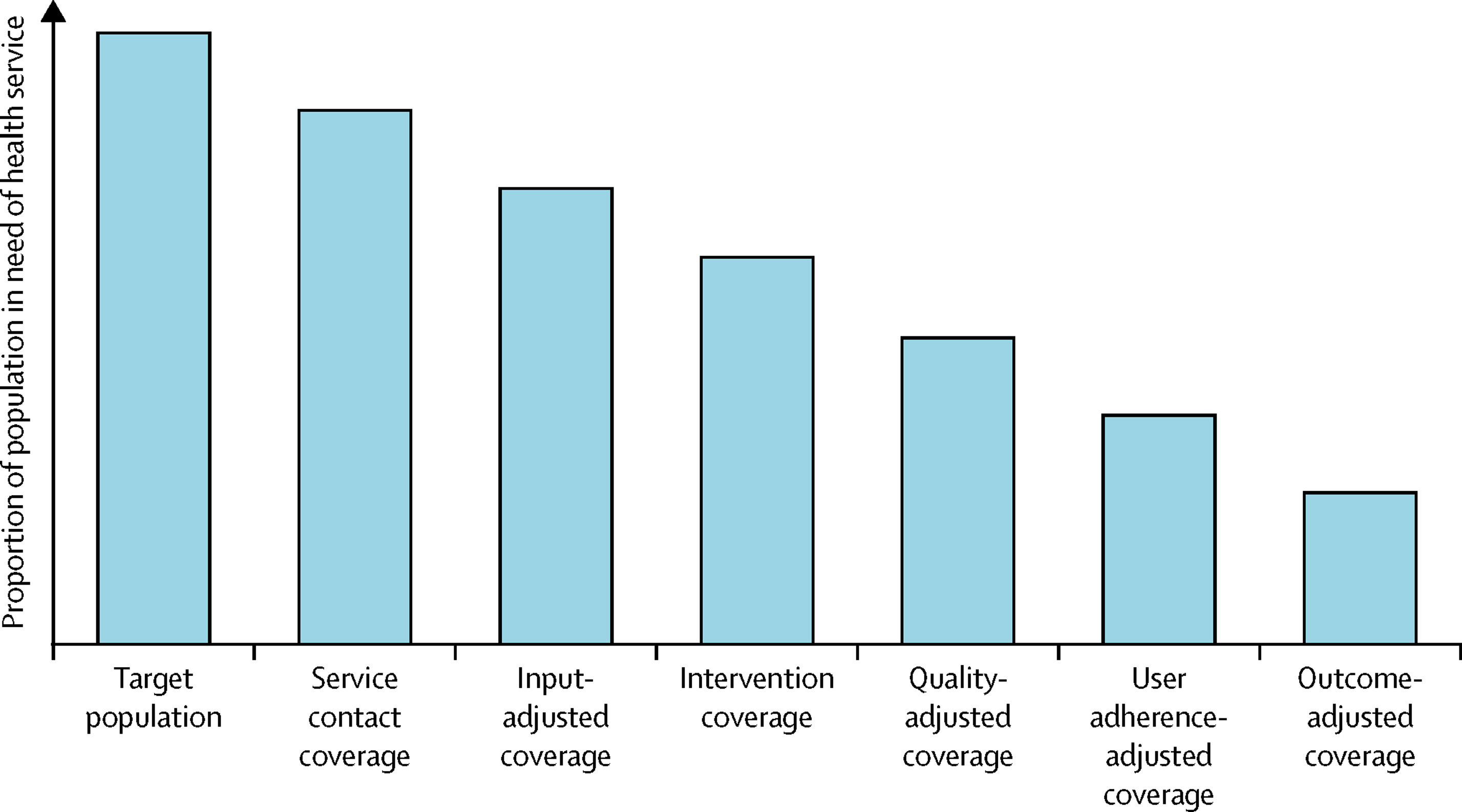


Supplementary Figure1: Effective coverage cascade (source: Marsh AD, Muzigaba M, Diaz T, et al. Effective coverage measurement in maternal, newborn, child, and adolescent health and nutrition: progress, future prospects, and implications for quality health systems The Lancet Global Health. Volume 8 Issue 5 Pages e730-e736 (May 2020))

Table S1: Reporting completeness of infrastructure and human resource items in health centres and hospitals by region, Ethiopia July 2022 June 2023

| Regions | Health Infrastructure | | | Human resources for health | | | Number of Health facilities |
| --- | --- | --- | --- | --- | --- | --- | --- |
|  | Electricity % | Sanitation facility % | Water supply % | Health Officer % | Midwife % | Nurse % |  |
| National | 54 | 61 | 48 | 62 | 67 | 68 | 4015 |
| Addis Ababa | 83 | 80 | 79 | 85 | 86 | 85 | 105 |
| Afar | 30 | 39 | 29 | 41 | 45 | 47 | 111 |
| Amhara | 64 | 73 | 56 | 78 | 82 | 82 | 972 |
| Benishangul | 20 | 29 | 26 | 30 | 37 | 37 | 76 |
| Central Ethiopia | 65 | 71 | 55 | 68 | 73 | 71 | 255 |
| Dire | 74 | 79 | 74 | 84 | 84 | 84 | 19 |
| Gambella | 18 | 18 | 3 | 24 | 24 | 26 | 34 |
| Harari | 92 | 92 | 92 | 83 | 67 | 75 | 12 |
| Oromia | 52 | 59 | 46 | 60 | 66 | 68 | 1565 |
| Sidama | 68 | 79 | 60 | 66 | 73 | 77 | 161 |
| Somali | 17 | 20 | 17 | 23 | 27 | 28 | 246 |
| South Ethiopia | 54 | 60 | 49 | 58 | 62 | 60 | 314 |
| Southwest | 58 | 63 | 41 | 66 | 72 | 73 | 145 |

Table S2. Percent of reporting completeness of DHIS2 for key data elements for ANC and skilled birth attendance by region, Ethiopia July 2022 -June 2023

|  | Antenatal Care | | | | | | Skilled Birth Attendance | | | | |
| --- | --- | --- | --- | --- | --- | --- | --- | --- | --- | --- | --- |
| Region | ANC1% | ANC4+  % | Hepatitis B tested  % | Syphilis tested  % | TD1  % | Iron folate  % | Skilled Birth Attendance % | Received  uterotonics % | Total live birth  % | Received chlorohexidine % | Live birth weighed % |
| National | 93 | 91 | 67 | 80 | 60 | 82 | 92 | 87 | 93 | 44 | 82 |
| Addis Ababa | 93 | 91 | 67 | 82 | 78 | 81 | 97 | 96 | 97 | 43 | 93 |
| Afar | 98 | 97 | 81 | 87 | 5 | 94 | 70 | 66 | 76 | 17 | 47 |
| Amhara | 95 | 92 | 71 | 85 | 75 | 82 | 98 | 96 | 98 | 52 | 93 |
| Benishangul | 97 | 96 | 77 | 91 | 89 | 90 | 64 | 55 | 68 | 18 | 60 |
| Central Ethiopia | 84 | 77 | 43 | 50 | 74 | 49 | 95 | 90 | 96 | 71 | 87 |
| Dire Dawa | 96 | 94 | 41 | 85 | 88 | 87 | 94 | 93 | 94 | 64 | 93 |
| Gambella | 94 | 91 | 45 | 60 | 73 | 78 | 61 | 59 | 65 | 5 | 39 |
| Harari | 80 | 74 | 39 | 43 | 70 | 56 | 99 | 99 | 99 | 72 | 79 |
| Oromia | 98 | 95 | 98 | 98 | 96 | 91 | 92 | 89 | 92 | 44 | 86 |
| Sidama | 70 | 63 | 36 | 44 | 42 | 59 | 96 | 93 | 96 | 68 | 90 |
| Somali | 76 | 54 | 27 | 35 | 71 | 54 | 75 | 57 | 82 | 12 | 41 |
| South Ethiopia | 94 | 93 | 93 | 94 | 93 | 93 | 93 | 85 | 95 | 35 | 79 |
| Southwest | 100 | 99 | 90 | 96 | 93 | 87 | 91 | 81 | 92 | 24 | 71 |

Table S3: Percent of DHIS2 reporting completeness of the 22 tracer drugs included in the input analysis in Ethiopia, July 2022 June 2023

| Tracer drugs | National | Oromia | Amhara | South Ethiopia | Central Ethiopia | Somali | Sidama | Southwest | Afar | Addis  Ababa | Benishangul | Gambella | Dire Dawa | Harari |
| --- | --- | --- | --- | --- | --- | --- | --- | --- | --- | --- | --- | --- | --- | --- |
| Glucose40 | 90 | 89 | 97 | 91 | 94 | 69 | 93 | 94 | 81 | 98 | 64 | 83 | 93 | 92 |
| Adrenaline injection | 90 | 89 | 97 | 91 | 94 | 69 | 93 | 94 | 82 | 98 | 64 | 84 | 93 | 92 |
| Amlodipine tablet | 90 | 89 | 97 | 91 | 94 | 69 | 94 | 94 | 82 | 98 | 63 | 84 | 93 | 92 |
| Amoxicillin dispersible suspension | 90 | 89 | 97 | 91 | 94 | 70 | 94 | 94 | 81 | 98 | 64 | 84 | 93 | 91 |
| Artemether Lumefantrine tablet | 90 | 89 | 97 | 91 | 94 | 69 | 94 | 94 | 81 | 98 | 64 | 83 | 93 | 91 |
| Ciprofloxcaxillin tablet | 90 | 89 | 97 | 91 | 94 | 69 | 93 | 94 | 81 | 98 | 64 | 83 | 94 | 92 |
| Cotrimoxazole240mg5ml | 90 | 89 | 97 | 91 | 94 | 70 | 94 | 94 | 81 | 98 | 64 | 84 | 93 | 92 |
| Frusemide tablet | 90 | 89 | 97 | 91 | 94 | 69 | 94 | 94 | 82 | 98 | 63 | 83 | 93 | 92 |
| Gentamycin injection | 90 | 89 | 97 | 91 | 94 | 70 | 94 | 94 | 81 | 98 | 64 | 84 | 93 | 92 |
| Hydralazine injection | 90 | 89 | 97 | 91 | 94 | 69 | 93 | 93 | 81 | 98 | 63 | 83 | 94 | 92 |
| Magnesium sulphate | 90 | 89 | 97 | 91 | 94 | 70 | 94 | 94 | 81 | 98 | 64 | 84 | 93 | 91 |
| Metformin tablet | 90 | 89 | 97 | 91 | 94 | 69 | 94 | 94 | 81 | 98 | 63 | 83 | 93 | 92 |
| Metronidazole capsule | 90 | 89 | 97 | 91 | 94 | 69 | 93 | 94 | 81 | 98 | 63 | 83 | 93 | 92 |
| NormalSaline09 | 90 | 89 | 97 | 91 | 94 | 69 | 93 | 94 | 81 | 98 | 63 | 83 | 93 | 92 |
| ORS-Zinc sulphate | 90 | 89 | 97 | 91 | 94 | 70 | 94 | 94 | 81 | 98 | 64 | 84 | 93 | 92 |
| Omeprazole capsule | 90 | 89 | 97 | 91 | 94 | 69 | 93 | 94 | 81 | 98 | 63 | 83 | 93 | 92 |
| Oxytocin injection | 90 | 89 | 97 | 91 | 94 | 70 | 94 | 94 | 81 | 98 | 64 | 84 | 93 | 92 |
| Pentavalent vaccine | 90 | 89 | 97 | 91 | 94 | 70 | 94 | 94 | 81 | 98 | 64 | 84 | 93 | 92 |
| RHZERH | 90 | 89 | 97 | 91 | 94 | 70 | 94 | 94 | 81 | 98 | 64 | 83 | 93 | 92 |
| TDF3TCDTG | 90 | 89 | 97 | 91 | 94 | 70 | 94 | 94 | 82 | 98 | 64 | 83 | 93 | 91 |
| TTC eye ointment | 90 | 89 | 97 | 91 | 94 | 70 | 94 | 94 | 82 | 98 | 64 | 84 | 93 | 91 |
| TAT | 90 | 89 | 97 | 91 | 94 | 69 | 93 | 94 | 81 | 98 | 64 | 83 | 93 | 92 |

Table S4: Steps and procedures to calculate effective coverage for ANC4+ service using DHIS2 data

| **Indicator name** | **Effective coverage of antenatal care (4 visits)** |  |
| --- | --- | --- |
| **Definition of the indicator** | **% of the population in need of quality antenatal care that received at least four antenatal care visits with quality** |  |
| **Target population:** | **All pregnant women** | **100%** |
| *Data source and reference period* | DHIS-2, from July 2022 to June 2023 |  |
| *Data element applied* | Number of pregnant women in the target population = number of ANC1 visit multiplied by percent of pregnant women who had at least one ANC from DHS 2019 report |  |
| **Contact coverage:** | % of pregnant women who came to the health facility for their 4^th^ ANC service | **48%** |
| *Data source and reference period* | DHIS-2 from July 2022 to June 2023 |  |
| *Data elements applied* | Total number of pregnancies by region; total number of ANC4 visits |  |
| *Method of generating contact coverage* | Number of pregnant women with at least ANC4 visit divided by total number of pregnant women |  |
| **Input-adjusted coverage:** | **% of pregnant women who seek care for their 4^th^ ANC in a health facility that is ready to provide the service.** | **24%** |
| *Data source and reference period* | DHIS2 and 2022-2023 |  |
| *Data elements applied* | **Infrastructure***:* Presence of electricity, water, and sanitation facility |  |
|  | **Essential medicine***:* Availability of 22 essential tracer drugs according to government list of essential drug (Glucose 40%, Adrenaline injection, Iron folate, Amlodipine tablet, Amoxicillin dispersible tablet, Arthmeter-Lumfanthrine, Cipro-floxacillin, Cotrimoxazole240mg5ml, Frusemide tablets, Gentamycin injection, Hydralazine injection, Magnesium sulphate injection, Metformin tablet, Metronidazole capsule, normal saline 09, ORS+/-Zinc sulphate, Omeprazole capsule, Oxytocin inj, Pentavalent vaccine, RHZE/RH, TDF/3TC/DTG, TTC eye ointment, Tetanus Anti-Toxin (TAT)) |  |
|  | **Human resource:** # of staff at the beginning of the year: nurse, midwife, health officer |  |
| *Other category (specify)* | None |  |
| *Method of generating input composite* | **Health Infrastructure:** percentage of facilities had at least one item; **Staffing**: % of facilities having 50% and above number of health workers and **Tracer drugs**: % of facilities with 50% and above percent of tracer drugs |  |
| **Intervention-adjusted coverage:** |  | **16%** |
| *Data source and reference period* | DHIS2 from July 2022 to June 2023 |  |
| *Data elements applied* | Tested for hepatitis B, tested for syphilis, received at least one TD vaccine and received iron folate |  |
| *Method of generating intervention composite* | Average of the percentages of the four interventions (tested for hepatitis B, for syphilis, received at least one TD vaccine and received iron folate) |  |
| **Process quality-adjusted coverage:** | **Not available** | **n/a** |
| *Data source and reference period* | n/a |  |
| *Data elements applied* | n/a |  |
| *Method of generating process composite* | n/a |  |
| **User-adherence-adjusted coverage:** | **Not applicable** | **n/a** |
| *Data source and reference period* | n/a |  |
| *Data elements applied* | n/a |  |
| *Method of generating process composite* | n/a |  |
| **Outcome-adjusted coverage:** | **Not applicable** | **n/a** |
| *Data source and reference period* | n/a |  |
| *Data elements applied* | n/a |  |
| *Method of generating process composite* | n/a |  |
| **Description of linking method applied, if any** | We took proportion of pregnant women with at least one ANC visit from the recent DHS in Ethiopia to estimate denominator as DHIS doesn’t provide target population size directly. |  |
| **Final effective coverage estimate** |  | **16%** |

Table S5: Steps and procedures to calculate effective coverage for skilled birth attendance service using DHIS2 data

| **Indicator name** | **Effective coverage of Skilled birth attendance** |  |
| --- | --- | --- |
| **Definition of the indicator** | % of the population in need of skilled birth attendance service with quality |  |
| **Target population:** | **All deliveries =Number of pregnant women multiplied by (1-pregnancy loss)**  **Pregnancy loss= 0.03** | **100%** |
| *Data source and reference period* | DHIS-2, from July 2022 to June 2023 |  |
| *Data element applied* | Number of skilled birth attendance, Number of deliveries |  |
| **Contact coverage:** | **% of deliveries attended by skilled birth attendant** | **62%** |
| *Data source and reference period* | DHIS-2 from July 2022 to June 2023 |  |
| *Data elements applied* | Total number of ANC1, percent of ANC1 coverage from DHS 2019, number of skilled birth attendance |  |
| *Method of generating contact coverage* | Number of skilled birth attendance divided by total number of deliveries |  |
| **Input-adjusted coverage:** | **% of pregnant women who seek for skilled birth attendance service in a health facility that is ready to provide the service.** | **30%** |
| *Data source and reference period* | DHIS2 and 2022-2023 |  |
| *Data elements applied* | **Infrastructure***:* Presence of electricity, water, and sanitation facility |  |
|  | **Essential medicine***:* Availability of 23 essential tracer drugs according to government list of essential drug (Glucose 40%, Adrenaline injection, Iron folate, Amlodipine tablet, Amoxicillin dispersible tablet, Arthmeter-Lumfanthrine, Cipro-floxacillin, Cotrimoxazole240mg5ml, Frusemide tablets, Gentamycin injection, Hydralazine injection, Magnesium sulphate injection, Metformin tablet, Metronidazole capsule, normal saline 09, ORS+/-Zinc sulphate, Omeprazole capsule, Oxytocin inj, Pentavalent vaccine, RHZE/RH, TDF/3TC/DTG, TTC eye ointment, Tetanus Anti-Toxin (TAT)) |  |
|  | **Human resource:** # of staff at the beginning of the year: nurse, midwife, health officer |  |
| *Other category (specify)* | None |  |
| *Method of generating input composite* | **Health Infrastructure:** percentage of facilities had at least one item; **Staffing**: % of facilities having average and above number of health workers and **Tracer drugs**: % of facilities with average and above percent of tracer drugs |  |
| **Intervention-adjusted coverage:** | **% of pregnant women who received skilled delivery, received uterotonics injection right after birth and received chlorhexidine for their newborn in a health facility that is ready to provide the service** | **21%** |
| *Data source and reference period* | DHIS2 from July 2022 to June 2023 |  |
| *Data elements applied* | Uterotonics injection right after birth for the mother, application chlorhexidine for chord care |  |
| *Method of generating intervention composite* | Average of the percentages of the two interventions (percent of mothers who received uterotonics right after birth and percent of newborn for whom chlorohexidine was applied for chord care) |  |
| **Process quality-adjusted coverage:** | **% of pregnant women who received skilled birth attendance service and received uterotonics injection right after birth and received chlorhexidine for their newborn in a health facility that is ready to provide the service according to the quality standard** | **19%** |
| *Data source and reference period* | DHIS2 from July 2022 to June 2023 |  |
| *Data elements applied* | Newborn weighed |  |
| *Method of generating process composite* | Number of newborns weighed divided by the total live birth during the same period |  |
| **User-adherence-adjusted coverage:** | **Note applicable** | **n/a** |
| *Data source and reference period* | n/a |  |
| *Data elements applied* | n/a |  |
| *Method of generating process composite* | n/a |  |
| **Outcome-adjusted coverage:** | **Not applicable** | **n/a** |
| *Data source and reference period* | n/a |  |
| *Data elements applied* | n/a |  |
| *Method of generating process composite* | n/a |  |
| **Description of linking method applied, if any** | We took proportion of pregnant women with at least one ANC visit from the recent DHS in Ethiopia to estimate denominator as DHIS doesn’t provide target population size directly. |  |
| **Final effective coverage estimate** |  | **19%** |

Box S1: Data collection tool: key informant interview guide

| **Section 1: Background information about the interview and the key informant**   1. General Information    1. Date of interview:    2. Name of Interviewer:    3. Interview Starting time:    4. Ending time:    5. General Observations/comments: ___________________________________ 2. Background Information    1. Sex of the key informant__________    2. Level of Education______________    3. Name of Organization __________________    4. Position in the Organization: __________    5. Year of experience on quality of health care and measurement program or project _____    6. Would you describe what your responsibilities are regarding quality of care and measurement?   **Section 2: General questions about Effective coverage measurement (ECM)**   - 1. Would you tell us what you know about effective coverage measurement that is used to assess quality of health service for MNCH in the Ethiopian health system?      1. Since our last interview, what were the development (new initiative or interest around ECM) in measuring effective coverage for MNCH in your organization?   2. How would you assess the awareness of ECM among:   - high-level policymakers(national),   - subnational decision makers and   - frontline implementers     1. How does this awareness, or lack thereof, influence the progress of your efforts (ECM implementation)?     2. Are there any initiatives aimed at introducing and advancing the **concept** of ECM by your organization?   **Section 3: Participants’ understanding of the ECM method and the result**  **Instruction:** the result has to be sent to the key informant via email with the letter; during the interview ask the participants if they looked at the result, if they didn’t, run through the result with the key informants.   - 1. How did you see the result from the presentation?   2. Did you have any questions about the methods that were used? Which questions?   3. What did you find most interesting about the results? Why was that interesting to you?   4. What did you find difficult or controversial about the output? Why was that?   5. How do you think these results (effective coverage measurements) help your understanding of trend in MNCH service quality?   6. How do you see the usability of such result in decision making in the Ethiopian health system?   Probe: Which level of the health system benefit from such evidence? in what way?   - 1. How often do you believe analysis like these should be conducted and shared with decision makers?   2. What is your opinion about the data source (DHIS2) we used for ECM, how do you see it compared to other data sources?   Probe: Comment on acceptability, feasibility and usability   - What would be the anticipated challenges? - Do you recommend utilizing DHIS2 for effective coverage measurement? Probe: Why? Why not?   1. Generally, what recommendations do you have regarding data use to further advance ECM in Ethiopia?   2. Are there any other key issues that we have not covered so far? Do you have any questions for me? |
| --- |
