# Supplementary material for: Effective coverage of maternal, neonatal and child health services based on District Health Information System 2 (DHIS2) data in Ethiopia: a mixed-methods study
Source: BMJ Open. 2026 Jan 23;16(1):e098795. doi: 10.1136/bmjopen-2025-098795 (PMC12853494; doi:10.1136/bmjopen-2025-098795)
Supplement: Supplementary file 2 [file bmjopen-2025-098795.reviewer_comments.pdf]

PEER REVIEW HISTORY

BMJ Open publishes all reviews undertaken for accepted manuscripts. Reviewers are asked to complete a checklist review form and are provided with free text boxes to elaborate on their assessment. These free text comments are reproduced below.

ARTICLE DETAILS

Title (Provisional)

Effective coverage of maternal, neonatal and child health services based on District Health Information System (DHIS2) data in Ethiopia: a mixed methods study

Authors

Lemma, Seblewengel; Getachew, Fikreselassie; Achamyeleh, Hiwot; Tesfa, Anene; Yihun, Bantalem; Kassahun Gelaw, Solomon; Getachew, Theodros; Persson, Lars; Schellenberg, Joanna; Marchant, Tanya

---

VERSION 1 - REVIEW

**Reviewer**	**1**
**Name**	**Chauke, Lawrence**
**Affiliation**	**University of Witwatersrand Department of Obstetrics and Gynaecology, Obstetrics and Gynaecology**
**Date**	**17-Feb-2025**
**COI**	**None**

REVIEW: Effective coverage of maternal, neonatal and child health services based on DHIS2 data in Ethiopia: a mixed methods study

Manuscript number: bmjopen-2025-098795

Type of article: original research

Thank you very much for the opportunity to review this paper.

SUMMARY

The aim of this mixed method study was to assess the feasibility of using DHIS2 data to estimate effective coverage of selected maternal and child health indicators and to explore perceptions of key stakeholders regarding effective coverage measures derived from Ethiopian DHIS2 data. The motivation behind the study was the recognition of need to move beyond the common practice of assessing access to care to include quality dimensions to improve maternal and child health outcomes . In terms of the study's main findings, there were more data elements for maternal than child healthcare services and as a result, the authors were not able to assess most of the child healthcare quality assessment indicators. Of the maternal healthcare services, the researchers were able to estimate the intervention-adjusted coverage

of ANC4+ and the process-quality-adjusted coverage of skilled birth attendance (16% and 19% nationally respectively) however there was no data to assess measures of postnatal intervention and process-quality. Similar findings were reported for sick childcare and child nutrition indicators. While the key stakeholders acknowledged the importance of the DHIS2 data in informing healthcare planning and decisions, they expressed concerns regarding the appropriateness and adequacy of the data collection , calling for standardisation of data and data sources.

COMMENTS

General

The topic that the study addresses is of global important. Quality of healthcare has been identified by the Lancet Commission on Quality Health Systems in the era of Sustainable Development Goals amongst the major contributing factors of mortalities and disabilities globally and especially, in LMICs. The importance of moving beyond assessing access to care to include quality is therefore undebatable. The choice of DHIS platform is justified since the platform is increasingly being used to collect data in public healthcare systems in LMICs. The paper is scientifically sound and easy to follow.

Abstract: The Abstract gives an excellent summary of what the study is all about and contains all the key elements.

Introduction: The introduction demonstrates the researchers' familiarity with the research subject, identified the existing gaps and provides an excellent justification for the study.

Method: The method is well described , scientifically sound and aligned with the research aims/objectives. The statistical measures selected are appropriate.

Results: While some components of the results are difficult to follow (perhaps demonstrating the complexities of the concepts investigated), the section is well presented and aligned with the aims/objectives of the study.

Discussion: The discussion section is well presented and appropriate.

Conclusion: Appropriate and relevant.

References: Appropriate and relevant

The following comments are made to further improve the manuscript /bring clarity :

1. Can the authors please clarify the study period? There is mention of 2015 and July 2022 to June 2023

2. While one appreciate the lack of universal consensus on measures of EC, it would be helpful for the readers if the authors can clarify why they decided to use 50% as a minimum cut off value for some of the EC measures (e.g. 50% or more of recommended staffing and essential drug list) and only one of the three infrastructure elements (electricity, sanitation facilities, and water supply) .

Thank you

**Reviewer**	**2**
**Name**	**Nimako, Kojo**
**Affiliation**	**World Bank Group, Health, Nutrition and Population**
**Date**	**16-Aug-2025**
**COI**	**None**

This paper is a beneficial effort by the authors to demonstrate the potential to advance effective coverage as a standard for assessing health system performance. In the same breadth it reveals the limitations of current routine health information systems in many low and middle income countries, demonstrated here through the case of Ethiopia. While the global community has advocated for stronger health information for decades, this work shows the urgent need for major overhauls in the scope and capacity of health information systems across countries, especially in shifting towards patient-level data collection and reporting, most efficiently achieved through electronic medical records systems.

I share a few thoughts below for the authors' consideration.

While this analysis uses aggregated patient-level data, the results do no represent the aggregate of the individual experiences across the care cascade. This is a limitation that significantly impairs the interpretation of the results. Consider an example where 50% of a population receive delivery care in well-staffed facilities and 50% of the population are provided with uterotonics after delivery; we may compute that 25% of the population are meeting both coverage standards defined here. In reality, this estimate is very likely lower than 25% (but never more than 25%) and even could be 0% if the section of the population that received one service is wholly different from the section of the population that received the other service. As such the authors must provide guidance on interpreting the results. For skilled birth attendance for instance, we can say that **at most** 19% *of the pregnant population accessed a health facility for childbirth care where there were adequate inputs (electricity, sanitation, or water supply, where staff and essential drugs were present), received appropriate interventions (uterotonics immediately after birth and chlorhexidine for the cord care) and according to the quality standard (newborn weighed)"*

The authors indicate that the selection of indicators for the different steps in the cascades was strongly influenced by the availability of data elements in the DHIS, but the reasoning for choosing certain thresholds for some of the identified data elements is not clear. For e.g., it is not clear why having only 50% of the recommended staffing level or only electricity, but no water or sanitation, is considered adequate. The authors should kindly provide the process or reasoning for defining these thresholds.

Related to the challenge of subjective threshold definitions, the authors should consider multiple thresholds for the indicators that are not all-or-none—e.g., for the input elements, high, middle and low thresholds can be defined. To illustrate, a high threshold could be where all of water, sanitation and electricity are present, and facility has at least 90% of staffing requirements, and at least 90% of essential medicines; a middle threshold could be where at

least two of water, sanitation or electricity are present, and facility has at least 70% of staffing requirements, and at least 70% of essential medicines; and a low threshold could be where at least one of water, sanitation or electricity is present; and facility has at least 50% of staffing requirements, and at least 50% of essential medicines. Using multiple thresholds allows estimation of a range of possibilities, from the aspirational to the non-negotiable, and avoids straight-jacketing LMIC contexts to low quality targets.

The national estimates for effective coverage computed in this analysis are quite low, and a significant portion of the deficit is due to low coverage of the first step in the cascade, the target population. With the significant uncertainties in determining the target population, it would be helpful for the authors to highlight two estimates for each of the indicators, the full population estimate (as currently reported) and an estimate of adjusted coverage for the component of the population accessing health services (i.e., the contact population is the denominator). The latter estimate can be deduced from Figure 1, but inclusion in the narrative and reflecting on it in the discussion would be beneficial to the reader. Highlighting these two estimates has the benefit of parsing out what proportion of the effective coverage deficit is from non-utilization and what portion is from poor quality during contact with the health system.

Related to the estimation of the target population, the Countdown 2030 strategy used by the authors in defining this population is predicated on the assumption that "*the health service is used by nearly all women or children and reporting completeness is near 100%" (Countdown 2030)*. It is not clear that these criteria, especially the latter, were met; for example, reporting completeness for ANC1 from facilities in the sample is 93%. The authors should clarify how they dealt with this not insignificant level of missingness for defining the target population?

More broadly on missingness, the authors should be explicit in the methodology about how they dealt with missing data in the DHIS. E.g., they report that data completeness for the application of chlorhexidine on the newborn baby's cord was only 44% and go on to report that 44% of babies had chlorhexidine applied to the cord. Does this mean that they considered all missing data as not performed?

While this is a methods paper, the results need some deeper reflection in the discussion. The effective coverage estimates generated are quite low (16% and 19%, and as low as 1% in some regions). This is despite some of the thresholds used for the input indicator being quite generous. The paper deserves a reflection on this low level of quality; are they expected, are the regional differences consistent with prevailing knowledge, are the findings corroborated by other studies?

Minor points

Private facilities were not included in this analysis. This is an important methodological decision and should appear in the methodology section. At the moment it is only captured as a limitation of the study.

Table 2 shows aggregate results by region, but the text on Page 13, lines 28-30 suggests it is by facility. Kindly revise the narrative appropriately.

For skilled birth attendance, it is not clear why the authors opted for *weighing of the newborn* as the process standard since the interventions listed (e.g., uterotonics immediately after birth) can also be used as process indicators. Some justification or background for this decision would be beneficial for future researchers who would be looking to replicate the analysis.

In the methodology the authors should include a disaggregation of the key informants, e.g., there were 10 from the MoH and 5 from partner organizations.

**Reviewer**	**3**
**Name**	**Langkulsen , Uma**
**Affiliation**	**Thammasat University**
**Date**	**13-Oct-2025**
**COI**	**None**

Thank you for the opportunity to review the manuscript titled “Effective coverage of maternal, neonatal and child health services based on DHIS2 data in Ethiopia: A mixed methods study”. Overall, I have several concerns regarding the manuscript. Below, I outline specific areas where improvements are recommended:

Abstract

- Please specify the statistical methods used for the quantitative analysis.

- Ensure that the keywords do not repeat words from the title to improve searchability.

Introduction

- Page 6, line 11, please check whether the neonatal indicators were unintentionally omitted, as the title includes “neonatal”.

Methods

Study Setting

- In the phrase “population of more than 100 million people”, please specify the year to provide context for the population figure.

Effective Coverage Analysis Using DHIS2 Data Elements

- Please clarify whether the “4015 health facilities: 3638 health centres, 252 primary hospitals, 94 general hospitals, and 31 referral hospitals” represent the entire country.

- The phrase “across regions” could be further clarified in the Study Setting section, as readers may not be familiar with the administrative regions of Ethiopia.

Qualitative Interviews with Key Stakeholders

- Please clarify the inclusion and exclusion criteria for selecting participants.

Results

- Please review the content, as some paragraphs appear more appropriate for the Methods section.

- In Table 2, please confirm whether "2022-2023" refers to the period from July 2022 to June 2023.

Discussion

- Please expand the discussion on severe acute malnutrition in relation to child nutrition services, and consider including nutrition targets for maternal and child health to address the double burden of malnutrition in children.

- Please elaborate on SDGs, food security, and food sovereignty, particularly in relation to maternal, neonatal, and child health services.

VERSION 1 - AUTHOR RESPONSE

Reviewer: 1
Dr. Lawrence Chauke, University of Witwatersrand Department of Obstetrics and Gynaecology
Comments to the Author:
REVIEW: Effective coverage of maternal, neonatal and child health services based on DHIS2 data in Ethiopia: a mixed methods study
Manuscript number: bmjopen-2025-098795
Type of article: original research
Thank you very much for the opportunity to review this paper.
SUMMARY
The aim of this mixed method study was to assess the feasibility of using DHIS2 data to estimate effective coverage of selected maternal and child health indicators and to explore perceptions of key stakeholders regarding effective coverage measures derived from Ethiopian DHIS2 data. The motivation behind the study was the recognition of need to move beyond the common practice of assessing access to care to include quality dimensions to improve maternal and child health outcomes . In terms of the study's main findings, there were more data elements for maternal than child healthcare services and as a result, the authors were not able to assess most of the child healthcare quality assessment indicators. Of the maternal healthcare services, the researchers were able to estimate the intervention-adjusted coverage of ANC4+ and the process-quality-adjusted coverage of skilled birth attendance (16% and 19% nationally respectively) however there was no data to assess measures of postnatal intervention and process-quality. Similar findings were reported for sick childcare and child nutrition indicators. While the key stakeholders acknowledged the importance of the DHIS2 data in informing healthcare planning and decisions, they expressed concerns regarding the appropriateness and adequacy of the data collection , calling for standardisation of data and data sources.

COMMENTS
General
The topic that the study addresses is of global important. Quality of healthcare has been identified by the Lancet Commission on Quality Health Systems in the era of Sustainable Development Goals amongst the major contributing factors of mortalities and disabilities globally and especially, in LMICs. The importance of moving beyond assessing access to care to include quality is therefore undebatable. The choice of DHIS platform is justified since the platform is increasingly being used to collect data in public healthcare systems in LMICs. The paper is scientifically sound and easy to follow.
Abstract: The Abstract gives an excellent summary of what the study is all about and contains all the key elements.
Introduction: The introduction demonstrates the researchers' familiarity with the research subject, identified the existing gaps and provides an excellent justification for the study.
Method: The method is well described , scientifically sound and aligned with the research aims/objectives. The statistical measures selected are appropriate.
Results: While some components of the results are difficult to follow (perhaps demonstrating the complexities of the concepts investigated), the section is well presented and aligned with the aims/objectives of the study.
Discussion: The discussion section is well presented and appropriate.
Conclusion: Appropriate and relevant.
References: Appropriate and relevant
Response: We appreciate the thorough review and constructive feedback.
The following comments are made to further improve the manuscript /bring clarity:
1. Can the authors please clarify the study period? There is mention of 2015 and July 2022 to June 2023
Response: Apologies for the confusion which arose because we had written the manuscript to reflect the Gregorian calendar, while Ethiopia uses the Coptic calendar. The Coptic calendar year 2015(the fiscal year) corresponds to July 2022-June 2023 in the Gregorian Calendar. We have added this explanation to the manuscript (see page 10, paragraph one).

2. While one appreciate the lack of universal consensus on measures of EC, it would be helpful for the readers if the authors can clarify why they decided to use 50% as a minimum cut off value for some of the EC measures (e.g. 50% or more of recommended staffing and essential drug list) and only one of the three infrastructure elements (electricity, sanitation facilities, and water supply).
Thank you
Response: Thank you, we have now added a paragraph that elaborates the reason for using the current cut off value in the discussion session (page 27, line 16-23).

Reviewer: 2
Dr. Kojo Nimako, World Bank Group
Comments to the Author:
Please find my comments in the attached file.

This paper is a beneficial effort by the authors to demonstrate the potential to advance effective coverage as a standard for assessing health system performance. In the same breadth it reveals the limitations of current routine health information systems in many low and middle income countries, demonstrated here through the case of Ethiopia. While the global community has advocated for stronger health information for decades, this work shows the urgent need for major overhauls in the scope and capacity of health information systems across countries, especially in shifting towards patient-level data collection and reporting, most efficiently achieved through electronic medical records systems. I share a few thoughts below for the authors' consideration.
Response: We appreciate the thorough review and constructive feedback.

While this analysis uses aggregated patient-level data, the results do no represent the aggregate of the individual experiences across the care cascade. This is a limitation that significantly impairs the interpretation of the results. Consider an example where 50% of a population receive delivery care in well-staffed facilities and 50% of the population are provided with uterotonics after delivery; we may compute that 25% of the population are meeting both coverage standards defined here. In reality, this estimate is very likely lower than 25% (but never more than 25%) and even could be 0% if the section of the population that received one service is wholly different from the section of the population that received the other service. As such the authors must provide guidance on interpreting the results. For skilled birth attendance for instance, we can say that at most 19% of the pregnant population accessed a health facility for childbirth care where there were adequate inputs (electricity, sanitation, or water supply, where staff and essential drugs were present), received appropriate interventions (uterotonics immediately after birth and chlorhexidine for the cord care) and according to the quality standard (newborn weighed)"
Response: this is insightful, and it is true that as none of the values are linked to individual level data hence least reflect an individual's experience. We have now added a statement in the limitation session to reflect the same (page 28, line 5-7).
The authors indicate that the selection of indicators for the different steps in the cascades was strongly influenced by the availability of data elements in the DHIS, but the reasoning for choosing certain thresholds for some of the identified data elements is not clear. For e.g., it is not clear why having only 50% of the recommended staffing level or only electricity, but no water or sanitation, is considered adequate. The authors should kindly provide the process or reasoning for defining these thresholds.
Response: We agree that this is important and have added a paragraph in the Discussion section. We used these more pragmatic cuts off points to compensate for the incompleteness of the data which partly explain the low level of these variables by region. As reported in the literature there is little consensus on how to define the cut off values in general, as well as what is included in each step of the cascade to measure the component scores (Exley J. 2022). The technical group at the WHO echoed the same in their recent publication (Strong K., et.al 2024). (page 27, line number 16-23)
Related to the challenge of subjective threshold definitions, the authors should consider multiple thresholds for the indicators that are not all-or-none—e.g., for the input elements, high, middle and low thresholds can be defined. To illustrate, a high threshold

could be where all of water, sanitation and electricity are present, and facility has at least 90% of staffing requirements, and at least 90% of essential medicines; a middle threshold could be where at least two of water, sanitation or electricity are present, and facility has at least 70% of staffing requirements, and at least 70% of essential medicines; and a low threshold could be where at least one of water, sanitation or electricity is present; and facility has at least 50% of staffing requirements, and at least 50% of essential medicines. Using multiple thresholds allows estimation of a range of possibilities, from the aspirational to the non-negotiable, and avoids straight-jacketing LMIC contexts to low quality targets.
Response: We appreciate the preference to see more granular effective coverage analysis and in preparing the manuscript the team discussed many options similar to those suggested here. However, for each scenario we were faced with the problem, echoed by our key stakeholders, that the DHIS2 data in Ethiopia is not yet ready to precisely estimate the effective coverage cascade. As such, the primary purpose of this analysis is not to interpret the reported figures for decision making but to reflect on the feasibility of using DHIS2 data for effective coverage analysis. In this context we think it is prudent to present one possible set of definitions. (page 27, line 16-23).

The national estimates for effective coverage computed in this analysis are quite low, and a significant portion of the deficit is due to low coverage of the first step in the cascade, the target population. With the significant uncertainties in determining the target population, it would be helpful for the authors to highlight two estimates for each of the indicators, the full population estimate (as currently reported) and an estimate of adjusted coverage for the component of the population accessing health services (i.e., the contact population is the denominator). The latter estimate can be deduced from Figure 1, but inclusion in the narrative and reflecting on it in the discussion would be beneficial to the reader. Highlighting these two estimates has the benefit of parsing out what proportion of the effective coverage deficit is from non-utilization and what portion is from poor quality during contact with the health system.
Response: We do appreciate the reviewer's preference to see more granular analysis to drive decision making about quality improvements needed in the health system. However, we respectfully argue that the analysis presented here has a slightly different purpose, to focus on the readiness of DHIS2 for effective coverage analysis in Ethiopia. We worry that more detailed data analysis could lead to an incorrect conclusion about DHIS2 data completeness and quality.

Related to the estimation of the target population, the Countdown 2030 strategy used by the authors in defining this population is predicated on the assumption that "the health service is used by nearly all women or children, and reporting completeness is near 100%" (Countdown 2030). It is not clear that these criteria, especially the latter, were met; for example, reporting completeness for ANC1 from facilities in the sample is 93%. The authors should clarify how they dealt with this not insignificant level of missingness for defining the target population? More broadly on missingness, the authors should be explicit in the methodology about how they dealt with missing data in the DHIS. E.g., they report that data completeness for the application of chlorhexidine on the newborn

baby's cord was only 44% and go on to report that 44% of babies had chlorhexidine applied to the cord. Does this mean that they considered all missing data as not performed? While this is a methods paper, the results need some deeper reflection in the discussion. The effective coverage estimates generated are quite low (16% and 19%, and as low as 1% in some regions). This is despite some of the thresholds used for the input indicator being quite generous. The paper deserves a reflection on this low level of quality; are they expected, are the regional differences consistent with prevailing knowledge, are the findings corroborated by other studies?
Response: Data quality is one of the issues we discussed as one of the feasibility challenges. We reported on the data quality for each health indicator in the results, and later in the discussion, we have provided evidence from other literature on the same. Regarding the approach used to estimate our denominator, the recommendation by the Countdown was the most plausible as we didn't have recent census data. None of the access variables were 100% but given that sample surveys have lower and upper ranges of certainty in their confidence interval, it is probably the best source of data available from which to estimate a target population. However, we do agree with the reviewer that this has limitations and added a comment in the discussion. (page 27, paragraph one).
Minor points Private facilities were not included in this analysis. This is an important methodological decision and should appear in the methodology section. At the moment it is only captured as a limitation of the study.
Response: We acknowledge that and now added more description of that in the method section on page 10, paragraph 1.
Table 2 shows aggregate results by region, but the text on Page 13, lines 28-30 suggests it is by facility. Kindly revise the narrative appropriately.
Response: it is now corrected in the same line.
For skilled birth attendance, it is not clear why the authors opted for weighing of the newborn as the process standard since the interventions listed (e.g., uterotonics immediately after birth) can also be used as process indicators. Some justification or background for this decision would be beneficial for future researchers who would be looking to replicate the analysis.
Response: As there is no consensus on what should be or should not be included in each step, we were guided by the definition of an intervention as having a biological mechanism to improve health vs a process being a behaviour or action that could lead to better care.
In the methodology the authors should include a disaggregation of the key informants, e.g., there were 10 from the MoH and 5 from partner organizations.
Response: the numbers are now disaggregated and added in the same line, page 10, line number 22-23.

Reviewer: 3
Dr. Uma Langkulsen , Thammasat University
Comments to the Author:
Thank you for the opportunity to review the manuscript titled "Effective coverage of maternal, neonatal and child health services based on DHIS2 data in Ethiopia: A mixed methods study". Overall, I have several concerns regarding the manuscript. Below, I

outline specific areas where improvements are recommended:

Abstract
1. Please specify the statistical methods used for the quantitative analysis.
Response: Thank you, this is now specified in the abstract, as 'descriptive analysis'.
2. Ensure that the keywords do not repeat words from the title to improve searchability.
Response: We have now removed those that were in the title and added new keywords.
Introduction
3. Page 6, line 11, please check whether the neonatal indicators were unintentionally omitted, as the title includes "neonatal".
Response: This is now added in the introduction session (page 5, line number 5).
Methods
Study Setting
4. In the phrase "population of more than 100 million people", please specify the year to provide context for the population figure.
Response: Thank you, we have added a time dimension to clarify the sentence.
Effective Coverage Analysis Using DHIS2 Data Elements
5. Please clarify whether the "4015 health facilities: 3638 health centres, 252 primary hospitals, 94 general hospitals, and 31 referral hospitals" represent the entire country.
Response: We have now expanded page 11, paragraph one, to address this issue and to clarify that these health facilities are public health facilities from all regions except Tigray where internal conflict led to a loss of data.
6. The phrase "across regions" could be further clarified in the Study Setting section, as readers may not be familiar with the administrative regions of Ethiopia.
Response: We added a sentence to describe the administrative structure of the country. (page 7, first paragraph)
Qualitative Interviews with Key Stakeholders
7. Please clarify the inclusion and exclusion criteria for selecting participants.
Response: As described in the section, we used a snowball approach to identify informants who have expertise in the topic. We had exclusion criteria as described in the section, which could exclude those without the said expertise.
Results
8. Please review the content, as some paragraphs appear more appropriate for the Methods section.
Response: Thank you for this comment. We do understand the confusion; however, the paper in general is dealing with the feasibility of a method (which is the use of DHIS2 to estimate effective coverage) thus the results are heavily focused on developing the method used and what worked and what didn't. We have made minor edits throughout to try to strengthen the link between the three steps described in the Methods and the three components of the Results.
9. In Table 2, please confirm whether "2022-2023" refers to the period from July 2022 to June 2023.
Response: It is now edited to reflect the comment on the same place.
Discussion
10. Please expand the discussion on severe acute malnutrition in relation to child

nutrition services, and consider including nutrition targets for maternal and child health to address the double burden of malnutrition in children.

Response: Thank you for this comment. We understand the importance of the result for the specific programs assessed. However, the focus of this paper is on understanding and assessing the feasibility of using DHIS2 as a data source for calculating effective coverage estimates for health programmes that are the priority of the Ethiopian government - and therefore of interest to our key stakeholders. For this reason, we respectfully prefer not to provide programmatic explanations on the specific public health interventions and the public health problems targeted by these interventions. We also didn’t make any programmatic recommendation regarding the public health interventions assessed.

11. Please elaborate on SDGs, food security, and food sovereignty, particularly in relation to maternal, neonatal, and child health services.

Response: We appreciate this comment; however, we remain keen to retain the main focus of this paper, which is to provide methodological evidence on the feasibility of using DHIS2 for the effective coverage measurement, and it is important that the discussion aligns with the original aim of the paper.
